# Risk factors and clinical characteristics of recurrent ectopic pregnancy: A case–control study

**DOI:** 10.1111/jog.14253

**Published:** 2020-04-12

**Authors:** Xinyan Wang, Lu Huang, Yan Yu, Sheng Xu, Yucheng Lai, Wenjie Zeng

**Affiliations:** ^1^ Department of Gynecology, Zhejiang Provincial People's Hospital, Hangzhou Medical College People's Hospital Hangzhou China

**Keywords:** recurrent ectopic pregnancy, risk factors, salpingotomy

## Abstract

**Objective:**

To compare signs and symptoms between patients with recurrent ectopic pregnancies (REP) and primary ectopic pregnancies (PEP) and to identify potential risk factors of REP.

**Materials and Methods:**

Data from 2014 to 2016 were analyzed. The study included 81 women each diagnosed with REP and PEP with no recurrence of ectopic pregnancy (EP) before January 2019. Information, including historical factors and findings at presentation of both group were collected. Data were compared between the two groups. Associations between REP and the risk factors were analyzed by logistic regression.

**Results:**

The findings revealed that compared to the patients in the PEP group, REP patients had significantly lower education (*P* = 0.001), higher proportion of previous infertility (*P* < 0.001) and different methods of PEP treatment (*P* = 0.001). Clinical data of the last operation revealed significantly higher occurrences of pelvic and peritubal adhesions (*P* < 0.05). Further multiple regression analysis showed that lower educational background (odds ratio [OR] = 4.183 95% confidence interval [CI] 1.311–13.344 *P* = 0.016), nulliparity (OR = 12.312 95% CI 3.382–44.824 *P* < 0.001), history of salpingotomy (OR = 7.129 95% CI 1.022–49.748 *P* < 0.05) and abortion (OR for one abortion = 21.576, *P* = 0.001; OR for two abortions =36.794, *P* < 0.001; OR for three abortions or more = 119.013, *P* < 0.001) were significant risk factors for REP.

**Conclusion:**

Active education on contraception is required for patients with lower educational level and history of abortion. Different plans should be formulated for patients with EP. For EP patients wanting fertility, the risk between fertility preservation and REP needs to be evaluated as reproductive function cannot be pursued blindly while ignoring the risk of recurrence.

## Introduction

Ectopic pregnancy (EP) is one of the most common gynecological emergencies. The most common site is the fallopian tube, accounting for approximately 90%of all reported ectopic pregnancies.^1^ With progression in early diagnosis and management, mortality caused by EP has decreased sharply; however, ruptured ectopic pregnancy continues to be a significant cause of marternal death during the first trimester of pregnancy.^2^

Recurrent ectopic pregnancy (REP), as a long‐term complication of ectopic pregnancy, results not only in anxiety about losing the pregnancy but also negative effects on future fertility and health. The incidence of REP in the literature varies from 10–27%,^3^, ^4^ representing a 5–15 fold increase in the general population.^5^ The widely accepted risk factors for EP are tubal damage resulting from pelvic infection or previous adnexal surgery, smoking, and in vitro fertilization (IVF)^4^, ^6^; however, the risk factors for REP remain unclear. Although some studies reported that the treatment success of single‐dose methotrexate (MTX) was independently protective for REP,^5^ there remains a lack of effective means to predict or prevent REP. Therefore, the present study compared the signs and symptoms between patients with REP and primary EP (PEP) to identify the potential risk factors of REP.

## Materials and Methods

### Study design and patients

The study was conducted at Zhejiang Provincial People's Hospital. Data from January 2014 to December 2016 were used. All procedures performed in the study were in accordance with the ethical standards of the Ethics Committee of Zhejiang Provincial People's Hospital and with the 1964 Helsinki Declaration. During the study period, 1132 patients were diagnosed with EP and admitted to the hospital. The diagnosis and location of pregnancy were confirmed during the operation for EP patients who received surgical treatment. Among patients who received medical treatment, the diagnosis was confirmed by a combination of serum beta‐human chorionic gonadotropin (b‐hCG) level and transvaginal ultrasonography. After excluding those special EP such as scar, cervical, cornual, intraperitoneal, ovarian pregnancies, and so on, there were 898 patients. The study included 81 women diagnosed with REP as the case group and 81 women diagnosed with PEP with no recurrence of EP before Jan 2019 as the control group. The PEP group was matched to the REP at a ratio of 1:1 with respect to age at the initial EP (± 5 years) and gestational week (±7 days) in the same period. The women were not followed prospectively. The information collected for each patient included historical factors and findings at presentation. The historical factors included socio‐demographic characteristics (age, body mass index (BMI), marital status, educational background, occupation, smoking status), reproduction and gynecology history (abortion, parity, infertility and pelvic inflammation disease (PID), the mode of pregnancy including natural conception, in vitro fertilization‐embryo transfer (IVF‐ET), surgical history, contraceptive methods and treatment of the primary EP. The findings at presentation included symptoms, gestational week at admission, mean adnexal size, HCG level, intraoperative conditions, and treatment methods.

### Statistical analysis

The data were analyzed using IBM SPSS Statistics for Windows, version 22.0 (IBM Corp.). Shapiro–Wilk tests were used to assess the normality of the sample distributions. Means ± standard deviation were used to describe the variables with normal distributions; medians and interquartile ranges were used to describe the variables with skewed distributions, and frequencies and percentage were used to describe the categorical variables. Significance of the differences between the REP and control groups was assessed by Chi square test and Fisher's exact tests for categorical variables. Student's *t*‐test was used for the analysis of continuous variables with normal distribution and Mann–Whitney *U*‐test for analysis of continuous variables with skewed distributions. Multivariate logistic regression with stepwise selection was used to evaluate the influencing factors. Two‐sided *P*‐value < 0.05 indicated statistical significance.

## Results

The median interval between REP and previous ectopic pregnancy was 25 months (rang: 5 months to 13 years). The PEP follow‐up duration ranged from 26 to 55 months, with a median of 33 months. Most women in the REP group had only one previous EP; 17 patients had two previous EPs and three patients had three EPs. Table 1 outlines the sociodemographic characteristics of all participants. Patients with REP were more likely to have a lower educational background than those with a PEP (*P* = 0.001). In addition, they were more likely to have had a higher number of abortions (*P* < 0.001)and a history of infertility(*P* = 0.001) than those with a PEP. Moreover, the primary EP treatment differed significantly. No significant difference was observed between the cases and the controls with respect to marital status, occupation, history of PID and pelvic surgery, parity, smoking history and contraceptive methods.

**Table 1:** Patient characteristics and demographics

variable	REP (*n* = 81)	PEP (*n* = 81)	t/Z/c2	*P* value
Marital status			2.837	0.134
Unmarried	14 (17.3)	23 (28.4)		
Married	67 (82.7)	58 (71.6)		
Education attainment			11.788	0.001
High school and lower	70 (86.4)	51 (63.0)		
College or above	11 (13.6)	30 (37.0)		
Smoking history	1 (1.2)	0 (0)	Fisher's	1.000
PID history	3 (3.7)	0 (0)	Fisher's	0.245
Parity			2.516	0.153
0	40 (49.4)	30 (37)		
≥1	41 (50.6)	51 (63)		
No. of prior abortion			39.471	<0.001
None	2 (2.5)	29 (35.8)		
One	22 (27.2)	25 (30.9)		
Two	22 (27.2)	18 (22.2)		
Three or more	35 (43.2)	9 (11.1)		
Contraception experience			Fisher's	0.709
None	51 (63)	51 (63)		
Condom	29 (35.8)	27 (33.3)		
Intrauterine device	1 (1.2)	3 (3.7)		
Mode of current pregnancy			Fisher's	0.120
Nature conception	77 (95.1)	81 (100)		
IVF	4 (4.9)	0 (0)		
History of infertility	10 (12.3)	0 (0)	10.658	0.001
Treatment of primary EP			17.584	<0.001
Expectant treatment	3 (3.7)	9 (11.1)		
Methotrexate	2 (2.5)	16 (19.8)		
Salpingectomy	24 (29.6)	14 (17.3)		
Salpingotomy	52 (64.2)	42 (51.9)		
History of pelvic surgery	16 (19.8)	8 (9.9)	3.130	0.120

EP, ectopic pregnancy; PEP, primary ectopic pregnancies; REP, recurrent ectopic pregnancies; IVF, in vitro fertilization.

The clinical characteristics and surgical findings of the two groups are shown in Table 2. No significant difference was observed between women with REP and those with PEP in initial HCG, diameter of accessory mass, and gestational week as well as symptoms such as bleeding, pain and others. However, we observed a significantly higher proportion of patients with pelvic adhesion or peritubal adhesions in the REP group than in the PEP group (*P* < 0.05).

**Table 2:** The clinical characteristics and surgical findings

	REP (*n* = 81)	PEP (*n* = 81)	Z/c2	*P*
Bleeding	51 (63)	61 (75.3)	2.893	0.125
Pain	38 (46.9)	44 (54.3)	0.889	0.432
Other symptom	15 (18.5)	7 (8.6)	3.366	0.107
Initial HCG	1644 (525–4077)	794 (323–3480)	−1.397	0.162
Diameter of accessory mass	2.7 (2–4.1)	2.7 (2–4)	−0.079	0.937
Days of amenorrhea	44 (39–48)	44 (37–50)	−0.159	0.873
Pelvic adhension & peritubal adhension	57 (76)	20 (35.1)	22.303	<0.001

HCG, human chorionic gonadotropin; PEP, primary ectopic pregnancies; REP, recurrent ectopic pregnancies.

Results of the multivariable analysis are listed in Table 3. Logistic regression analysis showed that the risk factors for REP were lower education (high school and below)(odds ratio [OR] =4.183, 95% confidence interval[CI] 1.311–13.344 *P* = 0.016), nulliparity (OR = 12.312, 95% CI 3.382–44.824 *P* < 0.001), salpingotomy (OR = 7.129 95% CI 1.022–49.748 *P* < 0.05) and abortion, with increasing ORs with the number of abortions(OR for one abortion = 21.576, *P* = 0.001; OR for two abortions = 36.794, *P* < 0.001, OR for three abortions or more = 119.013, *P* < 0.001).

**Table 3:** Multivariable logistic regression analysis predicting risk factors for recurrent ectopic pregnancies (REP)

	OR	95% CI	*P*
Education background (high school and below)	4.183	1.311–13.344	0.016
Nulliparous	12.312	3.382–44.824	<0.001
Number of abortion (*n* = 1)	21.576	3.593–129.569	0.001
Number of abortion (*n* = 2)	36.794	5.632–240.365	<0.001
Number of abortion (*n* = 3)	119.013	16.54–856.359	<0.001
Salpingotomy	7.129	1.022–49.748	0.048

## Discussion

In this case–control study, we found that compared to patients in the PEP group, REP patients had significantly lower education, higher proportion of previous infertility and differences in primary EP treatment. The findings at presentation showed no difference in clinical characteristics between the two groups, whereas the REP group had a higher proportion of patients with pelvic and peritubal adhesions. Multiple regression analysis showed that lower educational background, nulliparity, history of previous salpingotomy and abortion were significant risk factors for REP.

The incidence of REP reported in the literature varies with the study protocol, time interval between REP and previous EP in literature ranges from 4 months to 10 years, with an average of 2 years. The incidence of REP in our study was 9.02%, and the time interval was 5 months to 13 years, consistent with previous literature.

Correlation between previous infertility, PID, induced abortion and the occurrence of ectopic pregnancy is of great interest to researchers and has been well documented.^6^, ^7^, ^8^, ^9^ PID is one possible factor underlying reduced fertility and EP recurrence. Infertility caused by fallopian tube damage can be attributed to previous EP and surgical treatment. Patients with infertility have a greater chance of receiving surgical treatment or infertility work‐up, which also increases their risk of EP. Thus, the association between a history of infertility and REP could be due to tubal factors or the tubal damage caused by infertility treatment, which could, in turn, have caused EP.^10^ We observed a significant difference in previous infertility between the two groups; however, it was not a significant risk factor for REP. Although researchers have demonstrated a significantly increased hazard ratio for ectopic pregnancy in the subsequent pregnancy for abortion,^11^, ^12^ there have been few reports of abortion predisposing women to REP. While some studies have suggested no association between REP and history of abortion,^3^ others found that patients with recurrent EP were more likely to have had a spontaneous miscarriage, with increasing odds with the number of miscarriages. Our study has findings consistent with those of Butts *et al*.^4^, ^13^ that abortion was a significant risk factor for REP and that endometrial pathology or micro‐environmental hormonal milieu may play a common role in both conditions.^13^ After abortion, women are at increased risk of PID. Even in high‐income countries, the incidence of PID after abortion is as high as 10%^12^; however, the results of our study showed no significant difference in history of PID between the patients with REP and those with PEP. Although PID is not the key factor affecting the occurrence of REP, we found a significant higher proportion of REP patients with pelvic and peritubal adhesions, which may suggest under diagnosis of PID. Thus, from the perspective of REP prevention and treatment, active treatment of pelvic inflammation and reduction of the PEP can further reduce the incidence of REP.

Our study showed a significantly increased risk for REP in patients with lower educational levels. Hua *et al*.^14^ also reported that women's unemployment and low level of education increased the risk of EP after IVF‐ET. This finding may reflect a lack of knowledge about reproductive health in women with a lower educational background, which may lead to an increased risk of secondary infertility and EP or REP.

Our results also showed a significantly higher risk of REP in nulliparous women than in multiparous women, a finding consistent with that of a population‐based prospective cohort study conducted by Skjeldestad *et al*. in Norway.^3^ The fertility of women who have given birth confirms the normal functioning of their fallopian tubes, whereas some nulliparous conditions may be associated with infertility or abnormal fallopian tube function thus the risk of ectopic pregnancy is higher. The incidence of ectopic pregnancy in the general population is certain, and the reproductive demand of nulliparous is greater. With the increased possibility of pregnancy, the occurrence of EP or REP will inevitably increase.

As morbidity caused by EP has decreased, the clinical emphasis has now shifted fertility preservation. Proper management is essential. In addition to the effectiveness of the methods, both preserving fertility and reducing risk of recurrence must be considered.^15^ For tube EP, decision for medical or surgical management should be guided by the patient's clinical status, her desire for future fertility, and patient‐informed choice based on a discussion of the benefits and risks of each approach.^16^ Garbliu et.al showed that treatment success of single‐dose MTX was independently protective for recurrent EP.^5^ Randomized trails have demonstrated no difference in overall tubal preservation, tubal patency, EP recurrence or future pregnancies between medical management and tube‐sparing laparoscopic surgery.^17^ No significant difference in cumulative ongoing pregnancy rates have been reported between laparoscopic salpingotomy and salpingectomy^6^, ^15^, ^18^; however, the risk of REP according to the surgical technique remains controversial, with some showing no difference^19^, ^20^, ^21^ and others showing higher recurrence rates and higher rates of persistent trophoblasts after laparoscopic salpingostomy.^6^, ^18^, ^22^ Our study identified previous salpingotomy as a risk factor for REP. Women with history of EP are at risk for REP, with increasing risk with the number of previous EPs^6^, ^19^, ^23^, ^24^ as tubal damage resulting from salpingitis appears to be irreversible. If serious enough to cause an initial EP, the same intraluminal adhesions or dysfunction would continue to threaten any subsequent pregnancy.^25^ The shift in management strategies toward tubal conservation may contribute to increased recurrence. In rare cases such as cervical pregnancy and Cesarean scar pregnancy, early diagnosis is crucial for reducing the life‐threatening hemorrhage, preserving fertility, and ensuring survival. With advances in early detection of these rare cases, conservative treatment approaches such as local or systematic MTX injection followed by dilatation and curettage or hysterectomy removal of the gestational sac or treatment of uterine artery embolization(UAE) combined with MTX injection have good effect that help avoid hysterectomy and preserve fertility.^26^, ^27^

Recent findings suggest that women with ectopic first pregnancies have an increased risk of adverse birth outcomes during subsequent intrauterine pregnancies such as preeclampsia, preterm birth and emergency Caesarean delivery.^28^, ^29^, ^30^ It is not clear why women with EP may have an elevated risk of adverse birth outcomes in future pregnancies; however, several factors may contribute to this relationship. As advanced age is a risk factor of EP, older maternal age is also a risk factor for adverse outcomes. Risk factors such as previous pelvic surgery and endometriosis may also contribute to the association between EP and adverse birth outcomes in subsequent pregnancies and the surgery itself may be linked to the increased risk of adverse outcomes.^28^ Other than preserving fertility, we should consider the outcomes of future intrauterine pregnancies in patients with EP.

Our results indicate increased HRs of REP in women with a history of abortion and salpingotomy, a lower educational background, and nulliparity. Therefore, physicians should be aware of the clinical features of this condition. Different plans should be formulated for individual patients. Active education on contraception is necessary for patients with lower educational level and history of abortion to reduce unwanted pregnancies and REP. For EP patients wanting fertility, the risk between fertility preservation and REP needs to be evaluated as reproductive function cannot be pursued blindly while ignoring the risk of recurrence.

This study has several limitations. First, this is a retrospective study which carries inherent selective bias and information bias derived from the use of medical records. Second, we cannot exclude the possibility of future recurrence in patients in the PEP group due to the relatively short follow‐up period. Third, the mixture of patients with different treatments (surgical or medical) also carries a bias. Additional studies are needed with homogeneous treatments to reduce this bias. Fourth, this study had a relatively small sample size. Further prospective randomized controlled studies with larger sample sizes are needed to explore the optimal prevention methods in these patients.

## Conflict of Interest

The authors declare that they have no conflict of interest.
